# Resistance Evolution against Phage Combinations Depends on the Timing and Order of Exposure

**DOI:** 10.1128/mBio.01652-19

**Published:** 2019-09-24

**Authors:** Rosanna C. T. Wright, Ville-Petri Friman, Margaret C. M. Smith, Michael A. Brockhurst

**Affiliations:** a Department of Biology, University of York, York, United Kingdom; b Department of Animal and Plant Sciences, University of Sheffield, Sheffield, United Kingdom; University of California, Irvine; Georgia Institute of Technology School of Biological Sciences

**Keywords:** *Pseudomonas aeruginosa*, bacteriophage therapy, bacteriophages, evolutionary biology, resistance evolution

## Abstract

Phage therapy is a promising alternative to chemotherapeutic antibiotics for the treatment of bacterial infections. However, despite recent clinical uses of combinations of phages to treat multidrug-resistant infections, a mechanistic understanding of how bacteria evolve resistance against multiple phages is lacking, limiting our ability to deploy phage combinations optimally. Here, we show, using Pseudomonas aeruginosa and pairs of phages targeting shared or distinct surface receptors, that the timing and order of phage exposure determine the strength, cost, and mutational basis of resistance. Whereas sequential exposure allowed bacteria to acquire multiple resistance mutations effective against both phages, this evolutionary trajectory was prevented by simultaneous exposure, resulting in quantitatively weaker resistance. The order of phage exposure determined the fitness costs of sequential resistance, such that certain sequential orders imposed much higher fitness costs than the same phage pair in the reverse order. Together, these data suggest that phage combinations can be optimized to limit the strength of evolved resistances while maximizing their associated fitness costs to promote the long-term efficacy of phage therapy.

## Abstract (precis)

Globally rising rates of antibiotic resistance have renewed interest in phage therapy where combinations of phages have been successfully used to treat multidrug-resistant infections. To optimize phage therapy, we first need to understand how bacteria evolve resistance against combinations of multiple phages. Here, we use simple laboratory experiments and genome sequencing to show that the timing and order of phage exposure determine the strength, cost, and mutational basis of resistance evolution in the opportunistic pathogen Pseudomonas aeruginosa. These findings suggest that phage combinations can be optimized to limit the emergence and persistence of resistance, thereby promoting the long-term usefulness of phage therapy.

## INTRODUCTION

Globally rising rates of resistance against all major classes of chemotherapeutic antibiotics have renewed interest in the therapeutic use of phages to treat bacterial infections (1, 2). However, like antibiotics, the evolution of bacterial resistance to phages could limit the long-term sustainability of phage-based treatments (3). Therefore, it is important to understand the evolutionary processes determining the emergence of bacterial resistance against phage infection. Phages initiate infection by binding to molecules on the bacterial cell surface in a process called adsorption, so a common form of resistance is to prevent adsorption by modifying the targeted surface molecule(s) that acts as a phage receptor(s) (4–6). This can involve mutations affecting the regulation or biosynthesis of the surface molecule, leading to loss or altered structure of the receptor (6–8). Because the targeted surface molecules usually serve other functions—for instance, motility, cell membrane integrity, or nutrient transport—their alteration or loss is frequently associated with fitness costs, which can limit the success of resistant mutants in the absence of phages (9, 10).

Resistance mutations against a single phage typically occur at high frequency, and due to phage killing of susceptible genotypes, these resistance mutations will rapidly sweep through the bacterial population (8). Consequently, phage therapy usually involves the application of combinations of multiple phages, which is believed to limit the emergence of resistance in addition to targeting a wider range of bacterial genotypes (11, 12). There are two potential explanations for why phage combinations could limit the evolution of resistance. First, resistance to multiple phages may require multiple resistance mutations to arise in the same cell (13, 14), which is likely to occur with very low probability. Second, due to their associated fitness costs, the accumulation of multiple resistance mutations could lead to substantial loss of fitness if costs interact additively or synergistically (9, 10, 15, 16), reducing the ability of resistant cells to persist when faced with competition by nonresistant cells.

The evolution of resistance against phage combinations could occur more readily if individual resistance mutations protected against more than one phage, a property called cross-resistance. Cross-resistance is most likely to be elicited between phages that target the same bacterial surface receptor, where resistance mutations causing modification or loss of the shared receptor prevent the adsorption of each of the phages (7, 17, 18). Consequently, it is likely that successful phage combinations will tend to be those including phages that target multiple, distinct cell surface receptors because this will limit the potential for cross-resistance (14, 18). Moreover, although generalist cross-resistance mutations in global transcriptional regulators protecting against phages binding distinct receptors have been reported, these mutations occurred at lower frequency and incurred much higher costs than those affecting only a single receptor (7, 18).

Application of multiple phages in combinations either simultaneously or sequentially could also affect the trajectory of resistance evolution (7, 17, 19). Previous experimental studies suggest that sequential exposure can be as effective as applying phages simultaneously, but it was not tested whether these treatments selected for qualitatively different resistance mutations (17). It is likely that resistances selected under sequential versus simultaneous phage exposure will differ: sequential exposure provides more opportunity to accumulate multiple, specific resistance mutations, whereas simultaneous exposure may be more likely to select for a single, generalist cross-resistance mutation. Additionally, phage exposure prior to antibiotic treatment has been shown to improve bacterial killing more than either simultaneous treatment or the reverse order (i.e., antibiotic first [20, 21]). This suggests that the order in which antibacterial treatments are applied can have important effects on their efficacy, but the effect on the evolution of resistance to phage combinations remains unclear.

Here, we test experimentally how the evolution, genetic basis, and cost of resistance against pairs of phages targeting either the same or different cell surface receptors (lipopolysaccharide [LPS] and type IV pilus) in the Pseudomonas aeruginosa PAO1 strain depends on the timing and order effects when phages are applied in one step (i.e., simultaneous exposure) or sequentially. We found that sequential exposure allowed accumulation of multiple phage-specific resistances provided by mutations in cell surface receptor targets of phage adsorption without imposing any trade-offs in the strength of resistance. The order of phage exposure determined the fitness costs of sequential resistance, such that certain sequential orders imposed much higher fitness costs than the same phage pair in the reverse order. In contrast, simultaneous selection for phage resistances resulted in weaker resistance to both phages compared to the strength of sequentially acquired resistance with similar fitness costs. This suggests that phage combination treatments can be rationally designed to limit the strength of evolved resistances and to maximize fitness costs associated with resistance.

(Parts of this work were presented as a thesis for Rosanna C. T. Wright at the University of York.)

## RESULTS

### Sequential resistance is rarely limited by a trade-off in resistance strength.

**FIG 1:**
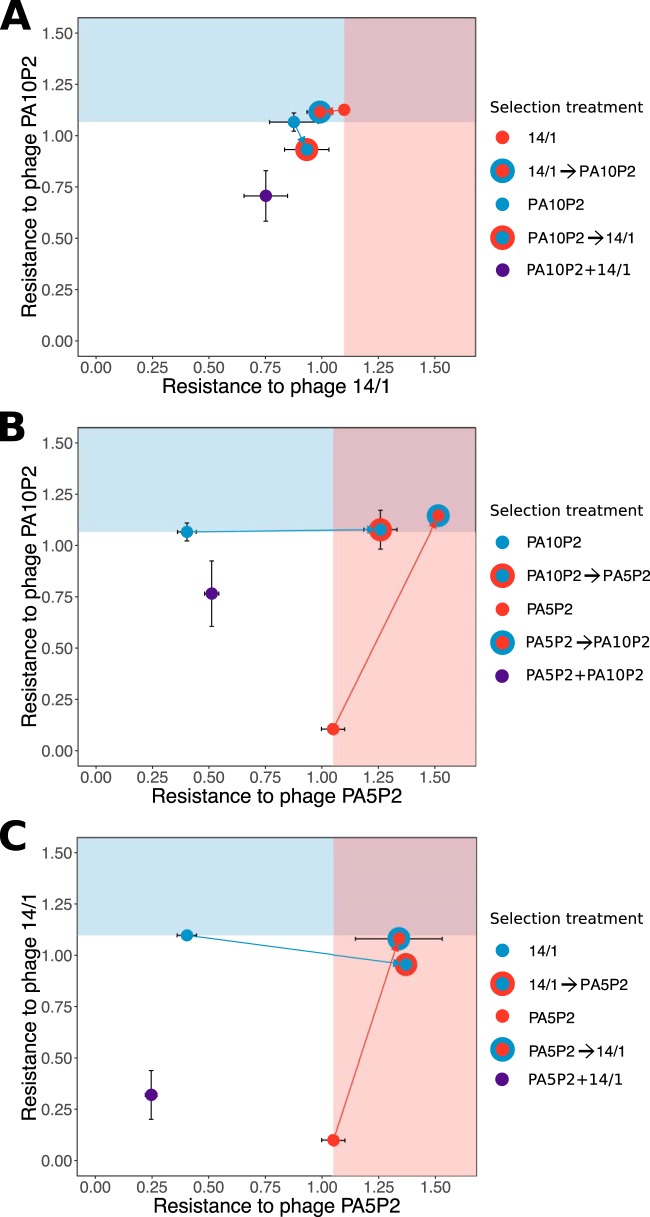
Trade-offs in the strength of resistance to each phage combination. Strength of resistance, measured as reduction in bacterial growth (RBG ± standard error [SE]), such that 1 indicates equal growth in the presence and absence of phage. Selection treatment for focal resistance is indicated by color (see individual keys). Background shading indicates the mean resistance strength for the first step in sequential resistance treatments to highlight changes in resistance to the first-step phage in the second step of sequential selection treatments.

By exposing the bacterial host to different phages sequentially, we could assess how the strength of resistance to each phage was influenced by the order of phage exposure. For a pair of phages targeting lipopolysaccharide (LPS) receptors (PA10P2 and 14/1), we typically found that the first-step resistance mutation provided resistance to both phages of approximately equivalent strength (Fig. 1A, Tukey test on analysis of variance [ANOVA] interaction, *P* > 0.99 in five out of six replicates). Hence, sequential selection for second-step resistance to the alternate LPS-associated phage did not tend to increase resistance against this second phage (Fig. 1A, Tukey test on ANOVA interaction, all *P* > 0.1). Moreover, after the second step of sequential selection, we observed only minimal trade-offs in resistance to the first phage (maximum decrease of ∼15%). Nonetheless, the order of exposure to phages appeared to have an effect on the occurrence of trade-offs: whereas no trade-off was observed in 14/1 resistance following second-step sequential selection of PA10P2 resistance (Fig. 1A, Tukey test on ANOVA interaction, all *P* > 0.1), PA10P2 resistance was slightly reduced following second-step sequential selection of 14/1 resistance in two out of three replicates (Fig. 1A, Tukey test on ANOVA interaction, mean differences of −0.140 and −0.259, both *P* < 0.001).

In contrast, for pairs of phages that targeted different receptors, that is either the LPS or the type IV pilus, the first-step resistance mutation rarely provided effective cross-resistance to the second-step phage. Whereas first-step resistance mutations against phage targeting LPS (PA10P2 or 14/1) provided weak cross-resistance to the type IV pilus-associated phage (PA5P2) in two out of six replicates (Fig. 1B and C, Tukey test on ANOVA interactions, *P* = 0.010 and 0.001), first-step resistance mutations against PA5P2 provided no cross-resistance against the LPS-binding phages. This suggests that, unlike for the phage pairs that shared the LPS receptor, multiple resistance mutations were required to protect against sequential phage pairs that targeted different receptors (Fig. 1B and C, Tukey test on ANOVA interaction, resistance to second phage after first step versus second step, all *P* < 0.001). However, the strength of the first-step resistance was rarely reduced following sequential selection for the second-step resistance (1 out of 12 replicates). Indeed, we observed increases in the strength of the first-step resistance in half of the replicates following acquisition of the second-step resistance mutation (Fig. 1B and C, Tukey test on ANOVA interaction, all *P* < 0.001). This implies that when phages target different receptors, sequential phage selection leads to additive resistances that rarely impose trade-offs in the strength of resistance to phages that adsorb to different cell surface receptors.

### Simultaneous phage selection results in weak resistance to both phages.

To determine whether equivalent resistances against pairs of phages could be achieved in one step rather than two sequential steps, we also selected resistant mutants against each phage pair simultaneously. Simultaneous selection by the phage pair targeting the LPS generally resulted in weaker resistance compared to the first-step and second-step resistance mutations observed during sequential selection with the same phages (Fig. 1A, Tukey test on ANOVA interaction, simultaneous selection weaker than first-step sequential selection in five of six replicates, *P* < 0.05; simultaneous selection weaker than second-step sequential selection in five of six replicates, *P* < 0.05). Similarly, simultaneous selection for resistance against the phage pairs targeting different receptors consistently resulted in weaker resistance to both phages compared to sequential resistance selection (Fig. 1B and C; Tukey test on ANOVA interactions, for PA5P2 resistance, all replicates weaker with *P* < 0.005; for PA10P2 resistance, three of six replicates weaker with *P* < 0.05; for 14/1 resistance, all replicates weaker with *P* < 0.05). Together, these results indicate that simultaneous exposure to multiple phages can reduce the strength of evolved resistance, even when the phage strains target the same receptor and promote reciprocal cross-resistance.

### Cost of resistance against multiple phages is determined by the order of applied phages.

**FIG 2:**
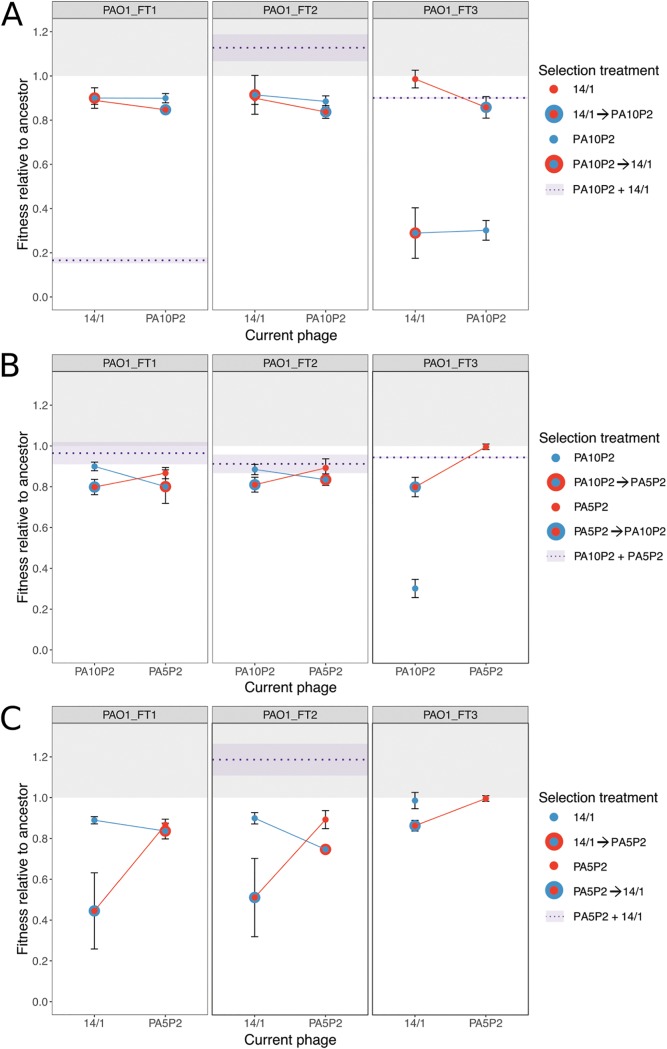
Relative fitness of resistant mutants is determined by selection regime. Fitness is measured as the maximum growth rate achieved during a 48-h growth period and given relative to that of the ancestral genotype; a value above 1 (light gray background) indicates lack of fitness costs associated with resistance mutation(s). Data points indicate the means of three technical replicates (±SE) measured for each set of biological replicates (plot headings indicate ancestral genotype used in the fluctuation test). Selection treatment for focal resistances is indicated by color (see individual keys). The mean relative fitness of simultaneous selection treatments is shown as a dashed purple line, with light purple shading to indicate standard error. Missing data indicate that no resistant mutants were recovered for that replicate treatment.

The emergence of highly resistant bacteria could be limited if strong resistance against multiple phages arising from sequential acquisition of resistance mutations was associated with higher costs (i.e., lower relative fitness). Thus, we next compared the growth kinetics of the resistant mutants relative to the phage-susceptible ancestral bacterial genotypes. Sequential resistance was more costly than equivalent simultaneous resistance in nearly half of replicates; however, we also observed that sequential resistance could impose equal or lower costs than equivalent simultaneous resistance (Fig. 2; Tukey test on ANOVA interactions, sequential resistance more costly in 6/13 replicates, *P* < 0.05; sequential resistance less costly in 2/13 replicates, *P* < 0.05; no significant difference in 5/13 replicates). This suggests that strong resistance against multiple phages can often be acquired sequentially without additional cost compared to weaker resistance acquired under simultaneous phage exposure. Nonetheless, order effects were important in determining the cost of resistance for some sequential resistances. When the first-step resistance was against a type IV pilus-binding phage followed by second-step resistance against a LPS-binding phage, we observed significant reductions in relative fitness following the second step in half of the replicates (Fig. 2B and C, Tukey test on ANOVA interaction, 3/6 replicates *P* < 0.05), whereas no significant change in fitness cost was observed with the opposite order of phage selection (i.e., exposure to first LPS-binding phage and then type IV pilus-binding phage). This suggests that the cost of resistance is mutation specific, and fitness can be determined by order effects indicative of epistatic interactions between specific resistance mutations.

### Genetic basis of phage resistance after sequential versus simultaneous phage selection.

To assess how the genetic basis of resistance varied between phage selection treatments, we sequenced the whole genomes of three resistant mutants exposed to each pairwise combination of the phages PA5P2, PA10P2, and 14/1 under both sequential and simultaneous selection regimes. Whereas single-phage resistance (i.e., first step of sequential selection) promoted acquisition of single mutations, selection of multiple phage resistances was generally provided by multiple resistance mutations in both simultaneous and sequential selection regimes (Tukey test on ANOVA interaction, second-step sequential selection versus first-step sequential selection, diff = 1.20, *P* < 0.0001; simultaneous versus first-step sequential, diff = 1.18, *P* < 0.001; simultaneous selection versus second-step sequential selection, diff = −0.018, *P* = 0.997).

**FIG 3:**
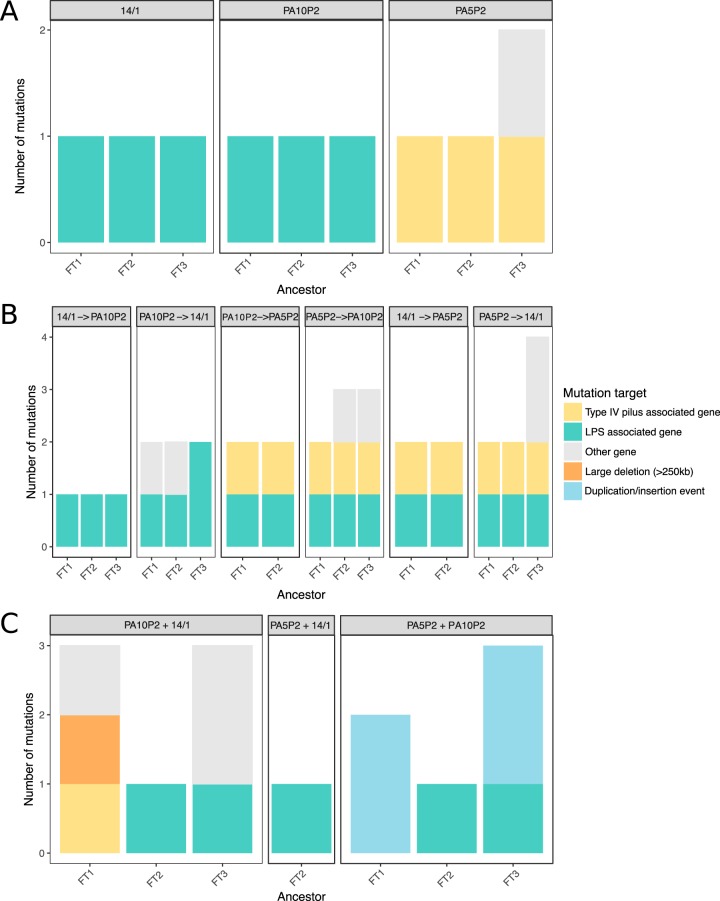
Treatment regimes determine the frequency and type of resistance mutations selected. Total number of identified mutations per resistant mutant across phage selection treatments (plot headings) under single (A), sequential (B), or simultaneous (C) selection. Colors indicate category of mutational target (see key).

For sequential resistance, we found that the first step resistance against all phages relied upon single variants in receptor-specific genes (Fig. 3A; see Table S1 in the supplemental material), with only one case of a secondary mutation which was likely due to hitch-hiking. Most mutations conferring first-step resistance to LPS-binding phages (PA10P2 and 14/1) targeted the *wzy* gene, which encodes a polymerase involved in synthesis of the LPS B-band O antigen (Table S1). All first-step resistance mutants selected using the type IV pilus-binding phage PA5P2 carried a single mutation affecting type IV pilus-associated genes (Fig. 3A; Table S1), including *pilB* (encoding a motor protein controlling pilus extension), *pilN* (encoding a product involved in pilus assembly), and *pilR* (encoding part of a two-component system regulating pilin production).

10.1128/mBio.01652-19.2 TABLE S1 Identity and function of genes where mutations were identified. Download Table S1, PDF file, 0.1 MB. Copyright © 2019 Wright et al. 2019 Wright et al. This content is distributed under the terms of the Creative Commons Attribution 4.0 International license.

Sequential resistance against combinations of phages targeting different receptors required a combination of two mutations, each affecting a distinct receptor-specific target (Fig. 3B; Table S1). Second-step resistance against the type IV pilus-binding phage PA5P2 required mutations targeting genes involved in pilus motility (*pilT* and *pilU*), extension and retraction (*pilB* and *pilY1*, respectively), and structural pilins (*pilE*) (note, however, that for two replicates we were unable to identify the first-step mutations and therefore these sequences were discarded). Second-step resistance against an LPS-binding phage (PA10P2 or 14/1) commonly required secondary mutations in *wzy*, the same gene that provided first-step resistance against these phages. Interestingly, however, in two out of three replicates of sequential selection with PA5P2 followed by 14/1, we observed second-step mutations in a distinct target, *galU*, a uridylyltransferase involved in LPS biosynthesis. Additional mutations in non-receptor-specific genes were found in three out of six replicates where resistance to the type IV pilus-associated phage was followed by sequential LPS-associated resistance, while no additional mutations were found in the reverse treatment order (i.e., LPS followed by type IV pilus) (Fig. 3B; Table S1). Taken together, these genetic data confirm that trade-offs in resistance strength between combinations of phages that bind different receptors were uncommon when resistance was acquired sequentially because multiple receptor-specific mutations were acquired, providing additive resistance.

**FIG 4:**
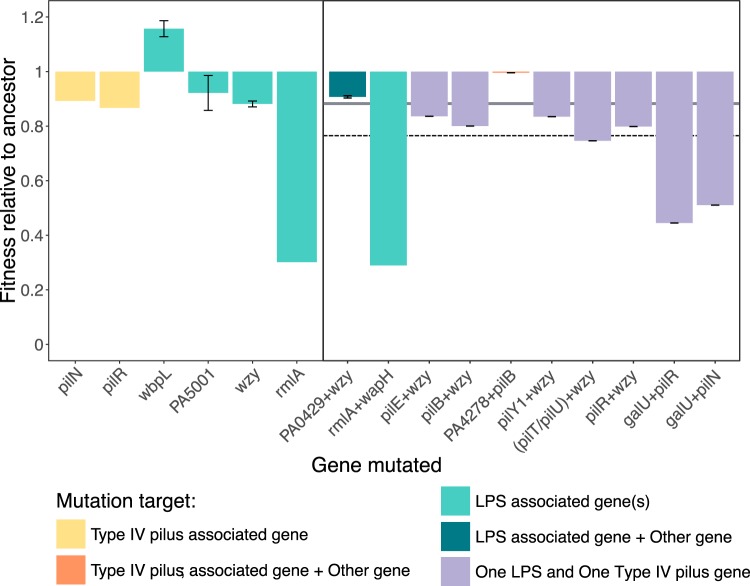
Contrasting fitness costs resulting from specific combinations of single and double mutations. Fitness relative to the ancestor, where a value of 1 indicates equal fitness and a value below 1 indicates a fitness cost associated with labeled single (left panel) or double (right panel) mutations. The dashed line indicates predicted additive cost of two mutations affecting different receptors, given as the sum of the mean cost of individual receptor-specific mutations (gray band; single LPS mutations RF = 0.886, single type IV pilus-specific mutations RF = 0.880).

In the case of sequential selection by pairs of phages targeting the same receptor, we observed order effects for the number and target of resistance mutations. Only one mutation was detected per replicate following sequential selection with phage 14/1 followed by PA10P2, whereas for the reverse order, we observed additional mutations in the second-step mutants (Fig. 3B; Table S1). For two replicates, we observed mutations in a gene of unknown function, PA0429, while the third replicate gained a second LPS-associated mutation (*wapH*, predicted glycosyl transferase; Table S1). It is unclear why parallel mutations in PA0429 would be selected because they provided no increase in resistance to 14/1 (in fact, the first-step resistance mutations in these replicates already provided strong cross-resistance to 14/1; Fig. 1), nor any reduction of the associated fitness costs (Fig. 4; Tukey test on ANOVA, *wzy* versus *wzy* plus PA0429, *P* = 0.978). In contrast, the *wapH* mutation did improve 14/1 resistance; here the first-step resistance mutation in the LPS biosynthesis gene *rml*, encoding a thymidylyltransferase, provided weak cross-resistance to 14/1 (Tukey test on ANOVA, strength of focal resistance versus cross-resistance, diff = −0.463, *P* < 0.0001), and furthermore, gaining the second-step resistance mutation was not associated with any increase in fitness costs (Fig. 4; Tukey test on ANOVA, *rmlA* versus *rmlA* plus *wapH*, *P* = 1).

In contrast, accumulation of multiple receptor-specific resistance mutations was never observed when phage pairs that targeted type IV pilus or LPS receptors were applied simultaneously (Fig. 3C). Half of the resistant mutants resulting from simultaneous exposure to phage pairs with different receptor targets carried mutations in LPS-associated genes without any additional type IV pilus-specific mutations (Fig. 3C). Surprisingly, we were unable to identify any mutations in two out of six replicates, suggesting that these clones had either displayed phenotypic resistance or had subsequently undergone a reversion mutation to restore phage sensitivity. In two resistance mutants, we observed duplication of the same two genes (approximately 1 Mb apart) both linked with DNA recombination: one encoding a single-strand DNA-binding protein (*ssb*) and the other a putative exonuclease (*PA3263*). However, the relationship between gene duplication and phage resistance is currently unclear.

Despite LPS-binding phages promoting strong reciprocal cross-resistance, two out of three resistance mutants selected against phages PA10P2 and 14/1 simultaneously had secondary mutations in non-LPS genes in addition to LPS-associated mutations. One mutant had a large deletion of approximately 250 kbp (around 4% of the whole genome), affecting more than 200 genes, including *galU*, the LPS biosynthesis gene that was also mutated under sequential selection against PA5P2 and 14/1 (Fig. 3C). This indicates that while PA10P2 and 14/1 both adsorb to the LPS, they probably target different sites, such that the strength of cross-resistance depends on specific effects of mutations on the LPS structure.

### Fitness costs associated with specific resistance mutations.

Across all genome-sequenced resistant mutants, those that had acquired multiple resistance mutations were less fit than those with only single resistance mutations (*t* test, *t*_93.2_ = 4.63, *P* < 0.0005). Fitness costs were often additive for combinations of LPS and type IV pilus mutations, with pairs of resistance mutations imposing greater costs than single resistance mutations on their own (Fig. 4). Second-step mutations in the LPS-associated gene *galU* increased fitness costs in combination with first-step mutations in type IV pilus genes (Fig. 4, Tukey test on ANOVA, *pilN* versus *pilN* plus *galU*, *P* < 0.0001; *pilR* versus *pilR* plus *galU*, *P* < 0.0001), but this was not the case for *wzy* which imposed no additional cost (Fig. 4, Tukey test on ANOVA, *pilR* versus *pilR* plus *wzy*, *P* = 0.826). Similarly, for sequential resistance selected against the pair of LPS-binding phages, secondary mutations in both LPS and non-LPS targets were effectively cost-free (Fig. 4, Tukey test on ANOVA, *wzy* versus *wzy* plus PA0429, *P* = 0.978; *rmlA* versus *rmlA* plus *wapH*, *P* = 1.00). While we were unable to calculate epistatic effects for most double mutants, as different loci were targeted in respective stages of sequential selection or multiple mutations were acquired concurrently, these results indicate that fitness costs of sequential resistance against multiple phages are likely to depend on epistatic interactions between the specific multiple resistance mutations.

## DISCUSSION

We compared the evolutionary trajectory of phage resistance selected sequentially versus simultaneously against pairs of phages targeting either the same or different cell surface receptors required for adsorption and infection of the bacterial cell. Sequential resistance to phage combinations targeting different receptors was acquired by accumulation of multiple receptor-specific mutations which promoted strong additive resistance to both phage strains without trade-offs in the strength of resistance to each phage. In comparison, simultaneous exposure to multiple phages resulted in weaker resistance to both phages through either single receptor-specific mutations or other major genetic variations (i.e., deletion and duplication events) affecting a larger number of genes. Furthermore, although sequentially acquired resistance was generally much stronger, it often imposed no greater cost to host fitness than the corresponding (weaker) simultaneous resistance. This suggests that simultaneous phage exposure imposes far greater constraint on resistance evolution than sequential exposure, leading to weaker resistance at the same fitness cost, which would be highly beneficial when applying phages in a therapeutic context.

Phages targeting the same receptor (LPS) provided strong cross-resistance to one another, such that the first step of sequential resistance provided equally strong resistance to both LPS-binding phages through acquisition of a single mutation affecting LPS biosynthesis, irrespective of the focal LPS-binding phage used to select resistance. We therefore expected that simultaneous and second-step sequential selection against two LPS-binding phages would be equivalent to the first step of sequential selection. Consistent with this, under sequential selection, resistance to both LPS-binding phages was provided by single mutations in LPS biosynthesis-associated genes, and although second-step mutations were observed upon second-step exposure to 14/1 (in PA0429), this mutation affected neither resistance or fitness. In contrast, under simultaneous exposure, multiple resistance mutations were observed in two out of three replicates, one of which was a large deletion affecting approximately 4% of the host genome. This suggests that even when phages target the same receptor, subtle differences in their target sites may make it harder to resist both phages simultaneously through a single mutation, even though single mutations providing resistance to both phages are available when encountered individually.

For combinations of phages targeting different receptors, we found that the order of exposure to phages could determine the trajectory of resistance evolution and its associated fitness costs. Sequential exposure to an LPS-binding phage following a type IV pilus-binding phage decreased the relative fitness of the host, whereas there was no significant change in the cost between the two stages of sequential exposure in the reverse order (i.e., type IV pilus-binding phage after LPS-binding phage). Similar type IV pilus mutations were selected in both sequential treatment orders, commonly affecting gene products involved in pilus-dependent motility [e.g., *pilB*, *pilT*, and *pilU* encode motor proteins, and *pilY1* encodes an anti-retraction factor [22, 23]). The LPS biosynthesis gene *wzy* (24) was targeted in both sequential treatments but imposed only modest increases in fitness cost, whereas mutations in *galU*, a uridylyltransferase also involved in LPS biosynthesis (25), were selected twice under selection with the LPS-binding phage 14/1 following initial selection against the type IV pilus-binding phage PA5P2 and caused severe decreases in bacterial fitness. Whereas mutations in *wzy* often affect only the B-band of LPS (13, 26), *galU* mutations may cause broader structural changes, affecting both the LPS core and A-band (27), which may explain their more detrimental effect on bacterial fitness. Additionally, it is possible that some primary mutations limit the acquisition of secondary mutations, for example, in second-step clones exposed to type IV pilus-binding phage PA5P2, we were unable to detect the LPS-associated mutations in *rmlA* and *ssg* present in the first-step sequenced clone (28, 29). Strong negative epistatic interactions between receptor-specific resistant mutations could limit or prevent the survival of bacteria carrying both mutations (although it should be noted that our experimental design did not permit direct measurement of epistatic interactions). Taken together, these data suggest that sequential selection with type IV pilus-binding phage followed by LPS-binding phage may both decrease the likelihood of multiple phage resistance genotypes arising and impose substantial fitness costs upon those cells that do evolve resistance, which may be sufficient to allow eradication of the infection by antibiotic treatment, or even by the patient’s own immune system. Rational design of sequential phage treatments to optimize these types of order effects could direct the trajectory of resistance evolution, allowing high fitness costs to be promoted and limiting fixation of certain resistance mutations.

Compared to sequential selection of resistance, simultaneous exposure promoted acquisition of weaker resistance. Whereas sequential resistances in different receptor targets provided additive resistance, simultaneous exposure often led to single receptor-specific mutations which provided relatively weak resistance to only one of the phages, or more-complex mutational effects (e.g., duplication and deletion events) targeting many genes, including some not known to be associated with LPS or type IV pilus. Although large deletions have been previously reported to provide phage resistance *in vitro* (30, 31), the loss of a large number of genes is likely to have major pleiotropic effects in more-complex environments where bacteria must respond to various environmental stressors (32, 33) and may be influenced by the resident microflora (34). Additionally, single mutations that do not provide resistance to all phages present in the cocktail may not be sufficient to allow survival under sustained phage pressure (e.g., if phage propagate *in vivo*). Overall, this suggests that there are fewer mutational routes allowing bacteria to survive in the presence of multiple phages simultaneously.

Bacterial phenotypic responses to stressors may also explain why we were unable to identify resistance mutations in some hosts exposed simultaneously to multiple phage strains. Stochastic environmental changes which affect bacterial lifestyle traits can hinder phage infection in numerous ways and create heterogeneity in host phenotypes within a population (35): changes in temperature can affect the rigidity of the host cell membrane, preventing phage infection (36), and nutrient variability can reduce expression of phage receptors (37–39). Changes in receptor expression levels could potentially limit phage adsorption sufficiently to enable survival of the host under simultaneous selection with multiple phage strains without leaving a genetic trace (40). Such resistances may be incidental to phage infection or induced by phage products in the environment and/or products of cellular lysis and may be considered phenotypic phage resistance (35, 37). Alternatively, survival in the presence of multiple phages may require such costly resistance mutations that as soon as phage selection pressures are relieved, reversion mutants restoring the original genotype rapidly outcompete the resistant genotype as the colony grows (41, 42). Although reversion is often much less likely than acquisition of compensatory mutations (43), we saw little evidence of compensatory mutations in our experiment; however, we did observe secondary mutations in genes of unknown function that arose concurrently with receptor-specific mutations, suggesting that compensation may be occurring. Ecological factors that influence population densities, adaptation responses, and stress-mediated effects may influence both the accumulation and persistence of phage resistance mutations by potentially promoting phenotypic resistance or rapid compensation; therefore, a better understanding of these interactions is required to design more durable phage therapeutics.

There are several caveats with respect to the wider implications of this work. First, we were largely unable to isolate resistant mutants against the pilus-binding phage PT7, suggesting that strong phage identity effects may overwhelm the effect of phage order and timing of exposure. It is possible that resistance mutations against PT7 are very rare or incur such high fitness costs that resistant mutants do not survive. Second, we consider here the emergence of spontaneous resistance mutations only during short <48-h incubations; different patterns of resistance could emerge over longer time periods, offering more opportunity for a wider range of mutations to arise, including compensatory mutations to ameliorate fitness costs. Third, we used only a limited number of phage strains targeting just two distinct bacterial receptors. To determine whether the principles of resistance emergence observed here can be generalized, future work will be needed to test a wider range of genetic and functional diversity of phages, including those binding a wider range of receptors, and to determine the contribution of other modalities of phage resistance in the bacterial hosts, for instance, CRISPR-Cas systems.

In conclusion, we found that the fitness cost of multiphage resistance could be maximized under specific orders of phage exposure by selecting for type IV pilus-associated resistance mutants followed by LPS-associated resistance mutants. As fitness costs are potentially exacerbated *in vivo*, due to more-intense competition for resources and the requirement of expression of a broader range of traits (44, 45), maximizing fitness costs associated with phage resistance could reduce the long-term success of resistant mutants. On the other hand, simultaneous exposure to multiple phage strains often imposed similar costs to sequential treatments but for weaker resistance against the phage combination. Due to the potential for phages to propagate and evolve to overcome bacterial resistance during treatment by replicating on susceptible bacteria, limiting the strength of evolved resistance may be just as beneficial as maximizing host fitness costs. Further experiments are warranted to determine the relative importance of these effects *in vivo*, as more-complex environmental pressures may exacerbate fitness costs and buffer differences in resistance strength. However, these results highlight that the rational design of phage cocktails to target multiple bacterial targets can limit the evolution of strong phage resistance and determine the fitness cost of evolved resistance.

## MATERIALS AND METHODS

### Strains and culture conditions.

Bacterial cultures of P. aeruginosa PAO1 were grown in King’s medium B (KB) as 6-ml volumes in a 30-ml glass microcosm with loose plastic fitting lids, and incubated at 37°C with shaking (180 rpm). Four genetically distinct phage strains with characterized adsorption targets (18) were used to select phage resistance: PA5P2 and PT7 (type IV pilus-binding phages) and PA10P2 and 14/1 (LPS-binding phages). Phage cultures were prepared directly from frozen glycerol stocks, added to approximately 10^7^ cells ml^−1^ of ancestral strain PAO1 from overnight culture and incubated overnight at 37°C with shaking (180 rpm). Phage stocks were purified by filtration to remove bacteria (0.22 μm) and stored at 4°C.

### Selection of spontaneous phage-resistant mutants.

We used a modified fluctuation test (46) to select spontaneous phage-resistant mutants against all pairwise phage combinations under both simultaneous and sequential selection. Three independent bacterial colonies were selected by streaking out the ancestral bacteria directly from glycerol stock onto KB agar (incubated overnight at 37°C); colonies were inoculated into 6 ml KB, incubated at 37°C (shaken) for 8 h, and then used to found 10 subpopulations by diluting each culture 10-fold into 10 wells of a 96-well microplate containing KB medium to a final volume of 200 μl. Samples of these initial bacterial cultures were also frozen (20% glycerol, −80°C) to be used for replicates and assigned the names PAO1_FT1, PAO1_FT2, and PAO1_FT3.

For each ancestral replicate, we selected phage resistance against each of the 4 individual phage strains independently (first step of sequential selection) and simultaneously against each possible phage pair (i.e., 6 different pairwise combinations) by exposing each of the 10 bacterial subpopulations to a different phage treatment. Bacterial subpopulations were diluted 100-fold into 600 μl of phage stock solution (300 μl of each phage for simultaneous selection) in deep-well 96-well plates, giving a multiplicity of infection of ∼100 phage per bacteria at a density of ∼10^7^ bacterial cells per ml, then plated out in 60-μl volumes onto KB agar. For combinations with low expected mutation frequency, the complete mixture was plated (i.e., all 600 μl in 60-μl volumes). Following incubation at 37°C for 24 h, single colonies were picked from each plate and restreaked onto KB agar to remove phage particles and incubated overnight. If no colonies were present, the same plates were incubated for a further 24 h at 37°C to allow slow-growing mutants time to produce colonies, which were then selected and restreaked as before. Single colonies were selected from streak plates and grown overnight in 6 ml KB, before glycerol stocks were prepared (20% glycerol, stored at −80°C) and characterized for phage resistance by cross-streaking against pure stocks of each phage strain on KB agar. For any phage treatments for which no bacterial colonies could be selected or where selected colonies lacked any phage resistance, the whole selection process was repeated using the same initial ancestral clone, inoculated directly from the glycerol stock to produce bacterial subpopulations. One clone per ancestral replicate was then chosen for further characterization (i.e., resistance strength, fitness costs, and sequence analysis), based on initial cross-streaking results. This produced a total of 12 resistant mutants selected against single phage strains (first step sequential selection – 3 ancestral replicates × 4 phage strains), and 18 resistant mutants selected against two phages simultaneously (i.e., 3 ancestral replicates × 6 pairwise phage combinations).

To complete sequential selection, we next exposed resistant bacteria selected against individual phages (i.e., first-step sequential selection) to each additional phage strain (second-step sequential selection). Clones selected in the first step of sequential selection were inoculated directly from glycerol stocks into 6 ml KB, grown for 8 h at 37°C, and then diluted 10-fold into 3 wells of a 96-well plate to found 3 subpopulations per clone, which were used to select second-step sequential resistance against each of the other phage strains using the same protocol as above. This produced a further 36 resistant mutants (3 ancestral replicates × 4 first-step phages × 3 second-step phages). As before, experiments with any clones lacking phage resistance or phage treatments producing no viable colonies were repeated. This was mainly an issue for combinations including phages PA5P2 and PT7 (both type IV pilus binding): up to 75% of populations yielded no resistant mutants, further compounded by a lack of “true” resistance in the selected colonies (up to 90% of selected colonies lacking quantitative resistance when selected against combinations including PT7 or PA5P2). Selection treatments including phage PT7 had extremely low mutation frequencies (e.g., 1 colony on >100 agar plates) and a high error rate both due to lack of phage resistance in selected PAO1 colonies and bacterial contamination (non-PAO1 colonies; this does not affect other treatments, as all resistant mutants presented were confirmed to be PAO1 using whole-genome sequencing). This indicates that true PT7 resistance is very difficult to acquire in combination with other phage strains, and therefore, treatments containing phage PT7 (under both sequential and simultaneous selection) were later discarded.

### Quantitative resistance assays.

To determine the strength of resistance provided by mutations selected at each stage of sequential selection and by simultaneous exposure to multiple phages, all spontaneous resistant mutants were assayed against all ancestral phage strains. Resistance assays were performed in 96-well microplates in KB medium added to a final volume of 150 μl. A ratio of approximately 10 phage per bacteria was used, giving densities of ∼10^7^ bacterial cells and ∼10^8^ phage particles per ml (i.e., multiplicity of infection of ∼10). The strength of resistance was measured as relative bacterial growth (RBG) in the presence of phage compared to RBG in the absence of phage (equation 1) (47) such that a value of 1 indicates complete resistance (i.e., equal growth in the presence and absence of phage). For resistant mutants with high fitness costs which result in slow growth (e.g., where the lag time may be up to 8 h), an endpoint of 48 h was used to calculate RBG instead of 8 h.

For phage *i*, bacteria *j*,

(1) $${\text{RBG}}_{\mathrm{ij}}=\frac{{\left[{\text{Abs}}_{600}\left(t=8\text{ h}\right)-{\text{Abs}}_{600}\left(t=0\text{ h}\right)\right]}_{\mathrm{ij}}}{{\left[{\text{Abs}}_{600}\left(t=8\text{ h}\right)-{\text{Abs}}_{600}\left(t=0\text{ h}\right)\right]}_{\text{control}j}}$$

where *t* denotes time and Abs_600_ indicates absorbance at 600 nm.

### Relative fitness of phage-resistant mutants.

To assess the fitness costs imposed by different resistance mutations, we compared the growth of all resistant mutants to the ancestral PAO1 strain in the absence of phage. Bacterial cultures were inoculated into 150 μl KB medium in 96-well microtiter plates directly from glycerol stocks and incubated at 37°C with a shaking cycle every 30 min when optical density was measured as absorbance at 600 nm for a total of 48 h to produce a growth curve. Four growth parameters were calculated from each growth curve to fully characterize changes in bacterial growth: the lag time before exponential growth begins, the maximum growth rate, the integral of the growth curve, and the maximum optical density reached (i.e., yield) (18). To calculate relative fitness, we used the maximum growth rate, as it correlated well with both the integral and maximum optical density (see Fig. S1 in the supplemental material). The maximum growth rate for each resistant mutant was divided by the mean ancestral maximum growth rate (with replicates matched within plates) to give relative fitness. A relative fitness of 1 indicates equal fitness with the ancestral strain, indicating that any resistance mutations present impose no fitness costs.

10.1128/mBio.01652-19.1 FIG S1 Multiple pairwise comparisons of growth curve metrics. The diagonal shows the distribution of each measurement extracted from 48-h growth curves of all phage-resistant mutants and phage-susceptible ancestral controls: maximum growth rate as change in absorbance through time (optical density at 600 nm [OD600]/h); lag time before exponential phage (h); yield (maximum OD600); and integral of the growth curve (OD600·h). Scatter plots in the lower quadrant give pairwise correlations with respective *R*^2^ values in the top quadrant. Download FIG S1, PDF file, 0.01 MB. Copyright © 2019 Wright et al. 2019 Wright et al. This content is distributed under the terms of the Creative Commons Attribution 4.0 International license.

### Sequence analysis.

All spontaneous phage-resistant mutants were sequenced using the Illumina MiSeq platform (performed by MicrobesNG, University of Birmingham), and resistance mutations were detected using the following bioinformatic analysis: short reads were aligned to an annotated PAO1 reference using Burrows-Wheeler Aligner (48), GATK Haplotype Caller (49) was used to identify single nucleotide polymorphisms (SNPs) and small indel variants, followed by annotation of gene information using SNPeff (50). Called variants were quality filtered by coverage (>20 reads per bp), and frequency of alternative allele (>80% of reads match alternative). Variants that occurred in all replicates were discarded, as they represent differences between the annotated PAO1 reference genome (GenBank accession no. or identifier [ID] AE004091) and the ancestral genotype used in our experiments. All replicates evolved from ancestral clone PAO1_FT3 contained the same SNP in gene PA3676; therefore, because this mutation must have arisen prior to the fluctuation test (i.e., when individual ancestral colonies were selected) and imposed no detectable fitness effect, it was ignored in subsequent analyses. Larger genetic variations, including deletions over 100 bp and duplication events, were identified by analyzing changes in coverage depth in R (51), and all variants were verified visually using an alignment viewer (52). Variation in the number of mutations acquired under different selection regimes was assessed using an analysis of variance (ANOVA) with post-hoc Tukey tests, including a main effect of ancestral replicate. The relative fitness of mutants carrying either single or double resistance mutations were compared using post-hoc Tukey tests following an ANOVA.

### Statistical analysis.

The effect of selection regime (i.e., simultaneous selection versus each stage of sequential selection) on both resistance and relative fitness data were analyzed using multiple ANOVAs (blocked by ancestral genotype and phage pair) followed by post-hoc Tukey tests. Multiple pairwise comparisons reported in the Results section were taken from the outputs of these models. Blocking by ancestral genotype and phage pair identity avoided violation of independence assumptions, i.e., so that differences between the ancestral genotypes (i.e., PAO1_FT3 has 1 SNP not present in PAO1_FT1 or PAO1_FT2) and resistance mutations from step 1 of sequential selection are accounted for in these analyses. In addition, the time at which RBG was calculated (i.e., if 48 h rather than 8 h for slow-growing resistant mutants with high fitness costs) was included as a random effect.

### Data availability.

Sequence data have been uploaded to the European Nucleotide Archive (accession IDs PRJEB33193 and PRJEB33233), and experimental data are available on Figshare (https://doi.org/10.15131/shef.data.9692753.v1).
