# Supplementary material for: Resistance Evolution against Phage Combinations Depends on the Timing and Order of Exposure
Source: mBio. 2019 Sep 24;10(5):e01652-19. doi: 10.1128/mBio.01652-19 (PMC6759759; doi:10.1128/mBio.01652-19)
Supplement: TABLE S1 [file mBio.01652-19-st001.pdf]

Category	Gene name	Operon/cluster	Gene product activity	Chromosome position (bp)	Effect type	Putative impact	Treatment	Ancestor
LPS biosynthesis	galU		UTP-glucose-1-phosphate uridylyltransferase	2215599-2215679	deleton (80bp)	HIGH	PA5P2->14/1	PAO1_FT2
				2215816	stop gained	HIGH	PA5P2->14/1	PAO1_FT1
	PA5001 (Ssg)	PA5005-PA5004-PA5003-PA5002-PA5001	cell-surface sugar biosynthetic glycosyltransferase	5618765	frameshift	HIGH	14/1<br>14/1->PA10P2	PAO1_FT3<br>PAO1_FT3
	PA5004 (wapH)	PA5005-PA5004-PA5003-PA5002-PA5001	probable glycosyl transferase	5622353-5622444	deletion (91bp)	HIGH	PA10P2->14/1	PAO1_FT3
	rmlA	rmlB-rmlD-rmlA-rmlC	glucose-1-phosphate thymidylyltransferase	5812961	missense variant	MODERATE	PA10P2<br>PA10P2->14/1	PAO1_FT3<br>PAO1_FT3
	wbpL	wbp cluster	glycosyltransferase	3529218	frameshift	HIGH	PA10P2+14/1<br>PA5P2+14/1	PAO1_FT2<br>PAO1_FT2
	wzy	wbp cluster	LPS B-band O-antigen polymerase	3538031	frameshift	HIGH	PA10P2<br>PA10P2->14/1<br>PA10P2->PA5P2	PAO1_FT2<br>PAO1_FT2<br>PAO1_FT2
				3538428	stop gained	HIGH	14/1<br>14/1->PA5P2<br>14/1->PA10P2	PAO1_FT1<br>PAO1_FT1<br>PAO1_FT1
				3538499	frameshift	HIGH	14/1<br>14/1->PA5P2<br>14/1->PA10P2<br>PA5P2->PA10P2	PAO1_FT2<br>PAO1_FT2<br>PAO1_FT2<br>PAO1_FT1
				3538988	frameshift	HIGH	PA10P2<br>PA10P2+14/1<br>PA5P2->14/1<br>PA5P2->PA10P2<br>PA5P2->PA10P2<br>PA5P2+PA10P2<br>PA5P2+PA10P2<br>PA10P2->14/1<br>PA10P2->PA5P2	PAO1_FT1<br>PAO1_FT3<br>PAO1_FT3<br>PAO1_FT2<br>PAO1_FT3<br>PAO1_FT3<br>PAO1_FT2<br>PAO1_FT1<br>PAO1_FT1
Type IV pilus biosynthesis	pilB		motor protein - pilus extension	5070925	missense variant	MODERATE	PA5P2<br>PA5P2->14/1<br>PA5P2->PA10P2<br>PA10P2->PA5P2	PAO1_FT3<br>PAO1_FT3<br>PAO1_FT3<br>PAO1_FT1
	pilE	fimU-pilV-pilW-pilX-pilY1-pilY2-pilE	minor pilin	5104842	conservative inframe insertion (12bp)	MODERATE	14/1->PA5P2	PAO1_FT1
	pilN	pilM-pilN-pilO-pilP-pilQ	type IV pilus assembly protein	5679190	missense variant	MODERATE	PA5P2<br>PA5P2->14/1<br>PA5P2->PA10P2	PAO1_FT2<br>PAO1_FT2<br>PAO1_FT2
	pilR	pilS-pilR	two-component response regulator	5095672	missense variant	MODERATE	PA5P2<br>PA5P2->14/1<br>PA5P2->PA10P2	PAO1_FT1<br>PAO1_FT1<br>PAO1_FT1
	pilT+pilU		twitching motility proteins	437058-438250	deletion (1,192bp)	HIGH	14/1->PA5P2	PAO1_FT2
	pilY1	fimU-pilV-pilW-pilX-pilY1-pilY2-pilE	putative anti-retraction factor	5101958	stop gained	HIGH	PA10P2->PA5P2	PAO1_FT2
				5103165	frameshift	HIGH	PA10P2+14/1	PAO1_FT1
Other gene variations	PA0142-nuh			163275	intergenic	MODIFIER	PA10P2+14/1	PAO1_FT3
	PA0429		predicted Nucleotide-diphospho-sugar transferase	480379	missense variant	MODERATE	PA10P2->14/1	PAO1_FT2
				480952	stop gained	HIGH	PA10P2->14/1	PAO1_FT1
	PA0845-PA0846		intergenic	923945	intergenic	MODIFIER	PA5P2->PA10P2	PAO1_FT2
	PA1065		unknown	1151442	missense variant	MODERATE	PA10P2+14/1	PAO1_FT3
	PA1567		predicted oxidation-reduction activity	1709197	conservative inframe insertion (6bp)	MODERATE	PA5P2->14/1	PAO1_FT3
	PA3722-PA3723			4167572	intergenic	MODIFIER	PA10P2+14/1	PAO1_FT1
	PA4278	birA-PA4279-PA4278	inferred sporutlation-related domain	4786538	missense variant	MODERATE	PA5P2<br>PA5P2->14/1<br>PA5P2->PA10P2	PAO1_FT3<br>PAO1_FT3<br>PAO1_FT3
Other				3651299-3651424	Duplication and insertion		PA5P2+PA10P2<br>PA5P2+PA10P2	PAO1_FT3<br>PAO1_FT1
				4746797-4747035	Duplication and insertion		PA5P2+PA10P2<br>PA5P2+PA10P2	PAO1_FT3<br>PAO1_FT1
				2194891-2445323	large deletion (>250 kb)		PA10P2+14/1	PAO1_FT1

Supplementary Table 1. Identity and function of genes where mutations were identified
