# Supplementary figures and images for: Resistance Evolution against Phage Combinations Depends on the Timing and Order of Exposure
Source: mBio. 2019 Sep 24;10(5):e01652-19. doi: 10.1128/mBio.01652-19 (PMC6759759; doi:10.1128/mBio.01652-19)

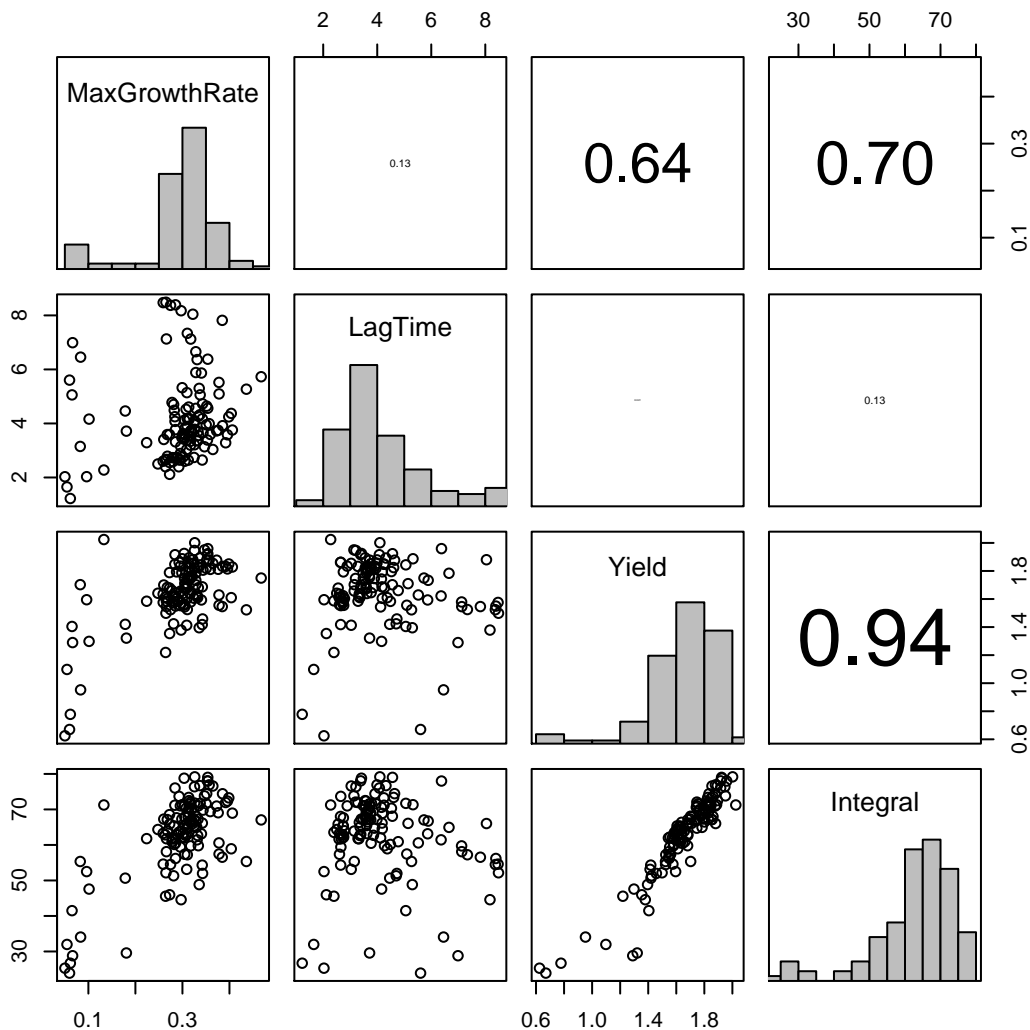

Supplement: FIG S1 [file mBio.01652-19-sf001.pdf]
